# Using graph-based consensus clustering for combining K-means clustering of heterogeneous chemical structures

**DOI:** 10.1186/1758-2946-5-S1-P50

**Published:** 2013-03-22

**Authors:** Faisal Saeed, Naomie Salim, Ammar Abdo, Hentabli Hamza

**Affiliations:** 1 Faculty of Computer Science and Information Technology, Universiti Teknologi Malaysia, Johor, Malaysia

## 

Consensus clustering methods are motivated by the success of combining multiple classifiers in many areas. In this paper, graph-based consensus clustering is used to improve the quality of chemical compound clustering by enhancing the robustness, novelty, consistency and stability of individual clusterings. For this purpose, Hyper-Graph Partitioning Algorithm (HGPA) [1], was applied. The clustering is evaluated based on the ability to separate actives from inactives molecules in each cluster and the results were compared with the Ward's clustering method. The chemical dataset MDL Drug Data Report (MDDR) database has been used for experiments.

The MDL Drug Data Report (MDDR) database consists of 102516 molecules. For the experiments, the dataset DS1 was chosen from the MDDR database. This dataset has been used for many virtual screening experiments [2-4]. The dataset DS1contains 10 heterogeneous activity classes (8568 molecules). For the clustering experiments, two 2D fingerprint descriptors will be used which are developed by Scitegic's Pipeline Pilot [5]. These are 120-bit ALOGP and 1024-bit extended connectivity fingerprints (ECFP_4).

The results were evaluated based on the effectiveness of the methods to separate actives from non-actives molecules using QPI- (for quality partition index) measure, which was devised by Varin et al. [6]. As defined by [7], an active cluster as a non-singleton cluster for which the percentage of active molecules in the cluster is greater than the percentage of active molecules in the dataset as a whole. Let p be the number of actives in active clusters, q the number of inactives in active clusters, r the number of actives in inactive clusters (i.e., clusters that are not active clusters) and s the number of singleton actives. The high value occurs when the actives are clustered tightly together and separated from the inactive molecules. Then the quality partition index, QPI, is defined to be:

(1) $$\text{QPI=}\frac{p}{p+q+r+s}$$

Then, the results will be compared with Ward's individual clustering method, the standard clustering method for chemoinformatics applications.

The generation process has been done by multiple run of K-means algorithms, each with random initialization of cluster centroids. The number of partitions generated in this step is ranged between n = 5 to n = 50, with 5-times step. Then, all the generated partitions were combined using HGPA to obtain the consensus partition. This process is done for each fingerprint (ALOGP and ECFP_4).

The mean of QPI values are averaged over the ten activity classes of the datasets. Tables 1, 2 show the effectiveness of MDDR dataset clustering using ALOGP and ECFP_4 fingerprints. The best PQI value of consensus clustering methods for each column has been bold-faced for ease of reference.

**Table 1:** Effectivenss of clustering of high diverse MDDR dataset: ALOGP Fingerprint.

Clustering Method			No. of clusters					
			
			500	600	700	800	900	1000
Consensus (HGPA)		N = 5	48.06	52.87	55.80	57.97	60.71	62.70
		N = 10	49.78	54.29	58.22	59.15	61.46	64.09
		N = 15	50.59	**55.20**	58.17	59.86	61.73	63.52
		N = 20	50.73	54.35	57.85	60.05	61.85	63.97
		N = 25	50.58	54.43	57.20	59.65	61.81	**64.16**
		N = 30	51.67	54.09	**59.26**	59.53	60.81	63.82
		N = 35	51.89	54.99	57.82	**60.80**	**63.14**	64.01
		N = 40	51.66	54.71	57.69	60.39	61.68	62.87
		N = 45	51.57	54.86	57.85	60.12	62.03	63.98
		N = 50	**52.44**	54.52	57.48	60.44	62.71	63.50
								
Individual	Wards' Method		39.01	41.83	44.49	46.03	47.89	49.45
								

**Table 2:** Effectivenss of clustering of high diverse MDDR dataset: ECFP_4 Fingerprint.

Clustering Method			No. of clusters					
			
			500	600	700	800	900	1000
Consensus (CSPA)		N = 5	57.36	61.39	65.25	68.93	71.90	74.69
		N = 10	58.51	64.01	67.98	70.23	75.04	75.79
		N = 15	61.28	64.45	68.16	71.27	73.44	74.34
		N = 20	60.78	64.92	**68.70**	71.22	74.37	74.45
		N = 25	62.03	65.88	68.46	71.11	**75.04**	74.27
		N = 30	61.85	64.64	67.27	70.17	73.35	**76.01**
		N = 35	**62.23**	**65.91**	68.44	71.30	72.97	73.75
		N = 40	61.67	64.62	67.79	69.31	73.61	74.92
		N = 45	61.80	65.11	67.96	**71.37**	74.07	75.41
		N = 50	60.91	64.96	68.56	70.57	74.57	73.33
Individual	Wards' Method		64.86	68.89	74.12	76.09	79.13	82.23
								

Visual inspection of the results enables comparisons to be made between the effectiveness of clustering of MDDR datasets and Ward's method, the best of choice clustering method for chemoinformatics applications. In addition, ten times of consensus clustering, for each fingerprint were observed in order to study the effectiveness of consensus clustering with different ensemble sizes. The results show that HGPA consensus clustering gives robust and novel result when K-means algorithm is run 20-50 times using ALOGP. The performance of consensus clustering outperforms the Wards' method.

For consensus of dataset which represented by ECFP_4 fingerprint, the best QPI values of consensus clustering are obtained from ensembles of size n = 20-50. The performance of consensus clustering gives robust results which are better than overall performance of individual clusterings. The values of QPI in both datasets for consensus clustering are close to the Wards method.

The consensus clustering, HGPA, provide stable clusters by decreasing the sensitivity to noise and outliers. The average percentages of singleton clusters of individual clusterings compared with consensus clustering for both fingerprints. The results show that consensus clustering partition the datasets with average percentage of singleton equal to zero, which is much better than individual clusterings and Wards' method. For example, 16.72% of molecules of DS1 are clustered as singletons when Wards method is applied on ALOGP fingerprint with number of clusters equal to 1000 clusters.

Finally we conclude that graph-based consensus clustering can improve the effectiveness of chemical compounds clustering. The performance of consensus clustering is more robust, novel, stable, consistent, and out-perform Wards' method in case of using ALOGP fingerprint. By using ECFP_4 fingerprint, consensus clustering methods provide more robust, stable, and consistent clustering and close to the Wards clustering results. The experiments reported here suggest that graph-based consensus clustering can improve the quality of individual clustering by using the efficient algorithm, K-means algorithm, to generate the ensemble with size (20-50) for both structurally diverse chemical datasets.
